# Molecular Dynamics Study on Properties of Hydration Layers above Polymer Antifouling Membranes

**DOI:** 10.3390/molecules27103074

**Published:** 2022-05-11

**Authors:** Heng Zhang, Jiyong Zheng, Cunguo Lin, Shiling Yuan

**Affiliations:** 1 Key Laboratory of Colloid and Interface Chemistry, Shandong University, Jinan 250199, China; zhangheng@sdu.edu.cn; 2 State Key Laboratory for Marine Corrosion and Protection, China Shipbuilding Industry 725 Research Institute, Qingdao 266071, China; zhengjy@sunrui.net (J.Z.); lincg@sunrui.net (C.L.)

**Keywords:** antifouling polymer, zwitterionic, surface hydration, molecular dynamics simulation

## Abstract

Zwitterionic polymers as crucial antifouling materials exhibit excellent antifouling performance due to their strong hydration ability. The structure–property relationship at the molecular level still remains to be elucidated. In this work, the surface hydration ability of three antifouling polymer membranes grafting on polysiloxane membranes Poly(sulfobetaine methacrylate) (T4-SB), poly(3-(methacryloyloxy)propane-1-sulfonate) (T4-SP), and poly(2-(dimethylamino)ethyl methacrylate) (T4-DM) was investigated. An orderly packed, and tightly bound surface hydration layer above T4-SP and T4-SB antifouling membranes was found by means of analyzing the dipole orientation distribution, diffusion coefficient, and average residence time. To further understand the surface hydration ability of three antifouling membranes, the surface structure, density profile, roughness, and area percentage of hydrophilic surface combining electrostatic potential, RDFs, SDFs, and noncovalent interactions of three polymers’ monomers were studied. It was concluded that the broadest distribution of electrostatic potential on the surface and the nature of anionic SO3- groups led to the following antifouling order of T4-SB > T4-SP > T4-DM. We hope that this work will gain some insight for the rational design and optimization of ecofriendly antifouling materials.

## 1. Introduction

The adsorption and accumulation of fouling organisms on surface of materials, i.e., marine biofouling, is a major problem faced by ships and offshore facilities [1,2]. The annual cost of increased fuel consumption, cleaning, maintenance, and repair of ships caused by marine biofouling is as high as billions of dollars [3,4]. Early marine antifouling coatings mainly used biotoxic tributyltin (TBT) antifouling paints, which killed marine organism larvae or spores through the release of antifouling agents to achieve antifouling purposes [5,6]. However, traditional antifouling paints are highly toxic for many aquatic organisms and have caused severe damage to the environment. The development of ecofriendly antifouling coatings is gradually becoming a research hotspot in this field [7,8,9].

Among them, protein-resistant antifouling material that inhibits the settlement of proteins is a relatively promising one [10], such as poly (ethylene glycol) (PEG), zwitterionic polymers [11] (poly (Sulfobetaine methacrylate), pSBMA, or poly (Carboxybetaine methacrylate), pCBMA). For example, Jiang’s group [12,13,14] has been engaged in biofouling research for a long period and synthesized a series of zwitterionic polymers. On the one hand, they used molecular simulation methods to reveal the antifouling mechanism of materials on the microscopic level. On the other hand, they carried out application research on this basis to design and synthesize new antifouling materials. Zheng and coworkers [15,16,17] investigated the antifouling properties of zwitterionic polymer brushes, polyacrylamide, and hydroxyalkyl acrylamides using combined molecular dynamics and steered molecular dynamics, believing that the carbon space and anionic groups have distinct effects on their antifouling performance. The state key laboratory of marine corrosion and protection in China has also synthesized a series of antifouling coatings by grafting zwitterionic sulfobetaine methacrylate (T4-SB) or anionic sulfonate methacrylate (T4-SP), which have the property of inhibiting adsorption of proteins on the surface of polysiloxane material (T4). These materials have a good antifouling effect on fouling organisms such as diatoms. We found that the static adsorption number of diatoms in the T4-SP antifouling material is 15/mm^2^ (4% of the T4 antifouling material) in the experiment; for T4-SB, the static adsorption number of diatoms is 9/mm^2^ (2% of the T4 antifouling material), which significantly improved the antifouling performance of the silicone material.

The adsorption of protein on surface is affected by many factors [18,19,20,21], among which the factors favorable for adsorption mainly include the enthalpy loss from the van der Waals and electrostatic attraction between protein and surface, and the entropic gain from the removal of hydration layer at the surface of material and protein. The disadvantages include the enthalpy gain required for the dehydration of surface and protein, protein’s conformation adjustment, as well as the entropic loss from protein adsorption and exposure of hydrophobic regions. The hydration layer above the surface of the antifouling material plays a crucial role from the antifouling perspective [22] because it provides the physical and energy barriers that must be overcome during protein adsorption. To confirm the structure of the hydration layer above the surface of antifouling materials, many experimental studies have been carried out. For example, Leng et al. [23,24] confirmed that there is a tightly bounded and regularly ordered hydration layer above zwitterionic antifouling membrane compared with polymer membrane without antifouling ability using sum frequency generation (SFG) vibrational spectroscopy. Paul et al. [25] directly observed the structure of hydration layer above the surface of epoxy organosilane modified silica nanoparticles and unmodified silica nanoparticles by frequency modulation−atomic force microscopy. Combined with molecular dynamics simulations, a more continuous and thicker hydration layer structure was found on the surface of modified silica particles, which endows the material with a better antifouling ability.

In this work, we will compare the antifouling ability of three polymer antifouling membranes (T4-DM, T4-SP, T4-SB) using molecular dynamics simulation at the molecular level through the hydration layer. We hope this work will provide theoretical support for the subsequent design and optimization of related antifouling materials.

## 2. Simulation Method

### 2.1. Model

**Figure 1:**
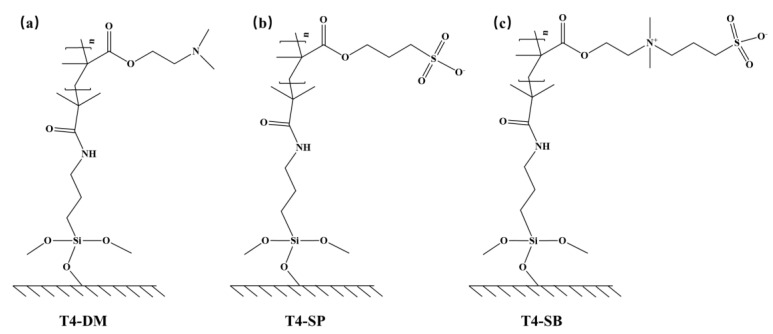
Chemical structure of three nonfouling membranes (**a**) T4-DM, (**b**) T4-SP, (**c**) T4-SB.

**Figure 2:**
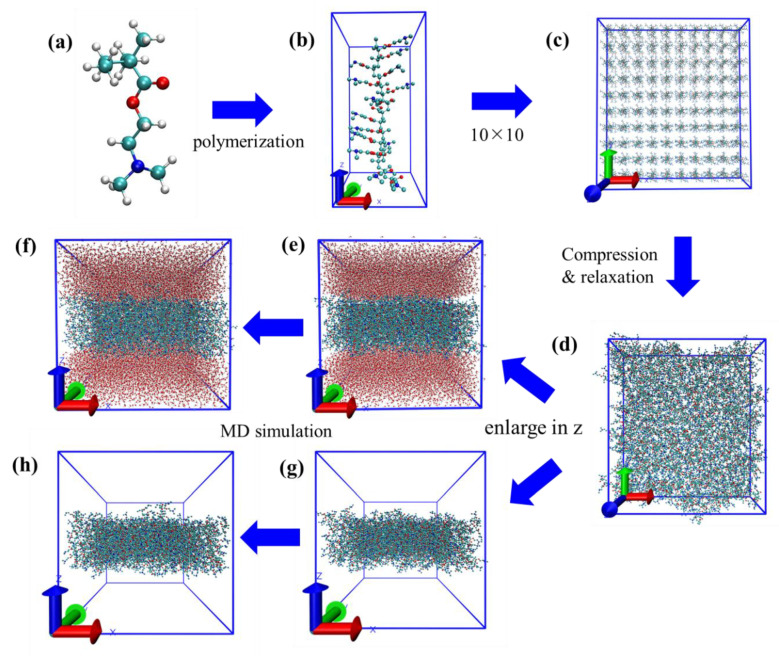
Modeling process and Simulation protocol of T4-DM system. (**a**) Repeat unit of DM; (**b**) single polymer chain of DM in simulation box (side view), (**c**) enlarged 10 times in x and y directions of (**b**) (top view); (**d**) compressed and relaxed configuration of DM membrane (top view); (**e**) initial configuration of DM with water system (side view); (**f**) final configuration of DM with water system (side view); (**g**) initial configuration of DM without water system (side view); (**h**) final configuration of DM without water system (side view).

Three antifouling membranes were constructed according to their molecular structures (Figure 1). The T4 substrate was neglected considering the main differences between different antifouling membranes focusing on the grafted polymers. The modeling process of T4-DM system is illustrated in Figure 2 as an example. The polymer chains with a degree of polymerization of 15 (Figure 2b) were built from their repeat unit (Figure 2a) using the Visualizer module in Materials Studio. This was repeated 10 times in the x and y directions to derive the initial configuration of antifouling membrane in Figure 2c. The initial configurations of the antifouling membranes were then subject to a 21-step molecular dynamics compression and relaxation [26] to obtain the equilibrium packing structure (which might not be the optimal one) in Figure 2d. The procedure of the 21-step MD simulation protocol is listed in Table S1. The simulation boxes were then enlarged two times along the z-axis to accommodate solvent molecules (Figure 2e). As a comparison, antifouling membranes without water were also studied (Figure 2g). Finally, all systems were subject to equilibrium molecular dynamics simulations to derive equilibrium structures (Figure 2f,h).

### 2.2. Simulation Details

The repeat unit of each polymer was calculated at B3LYP/def2SVP// B3LYP/def2TZVP level using Gaussian 16 [27]. Then, RESP charges were derived from Multiwfn 3.8 [28]. All molecular dynamics simulations were performed using Gromacs 2019.3 software package [29]. Gromos 54a7 force field was used [30]. The total potential energy was given as a combination of valence terms, including bond stretching, angle bending, torsion, and nonbonded interactions. The nonbonded interactions between atoms were described by the Lennard-Jones potential, and the standard geometric mean combination rules were used for the van der Waals interactions between different atom species. Water molecules used the SPC model [31]. 

In the simulations, each of the systems was initialized by minimizing the energies of the initial configurations using steepest descent method. Following the minimization, a 50 ns MD simulation under NPT ensemble was carried out for each system, with a time step of 2 fs. In all simulations, the temperature was kept constant at 298 K by the v-rescale thermostat algorithm [32]. The pressure was kept constant at 1 atm by the Berendsen algorithm [33]. Bond lengths were constrained using the LINCS algorithm and periodic boundary conditions were applied in all directions [34]. Short-range nonbonded interactions were cut off at 1.2 nm, with long-range electrostatics calculated using the particle mesh Ewald method [35]. Trajectories were stored every 2 ps and visualized using VMD 1.9.3 [36].

## 3. Results and Discussion 

### 3.1. Properties of Antifouling Membranes

#### 3.1.1. Density Profiles

The simulated configurations of three antifouling membranes at dry and hydrated states are illustrated in Figure S1. We can clearly see that there are no significant differences between T4-DM membrane under dry and hydrated states, while for T4-SP and T4-SB membranes, many side chains extend to water phase. This indicates that the side chains of T4-SP and T4-SB have a better hydrophilicity. Besides this, the compression of these chains during adsorption of foulant would reduce the conformation possibility, which is entropically unfavorable, subsequently causing steric repulsion and preventing adsorption [10].

**Figure 3:**
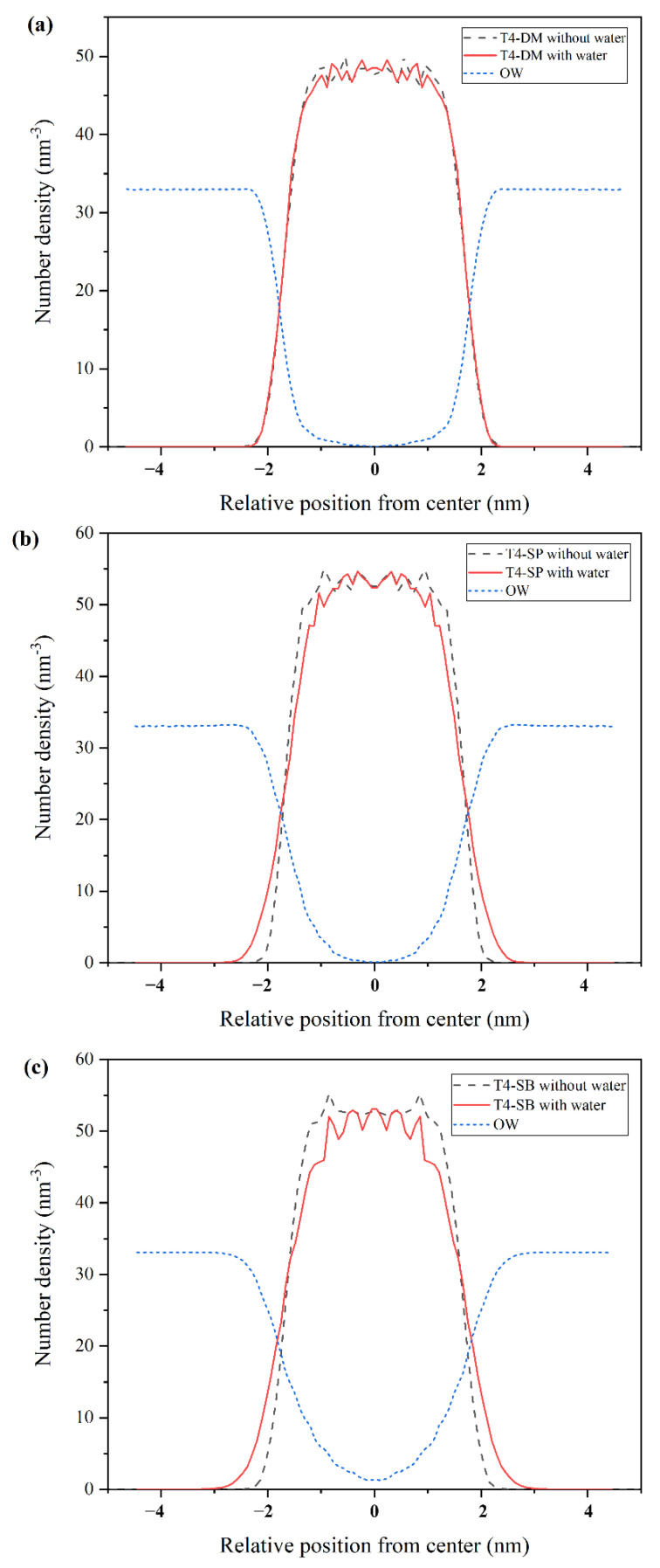
Density profiles along z-axis. (**a**) T4-DM, (**b**) T4-SP, (**c**) T4-SB. Black dashed lines and red lines represent density of antifouling membranes under dry state or hydrated states. Dotted blue lines represent density of water.

To quantitatively study the structure of three antifouling membranes, the density profile along z-axis was calculated, as shown in Figure 3. The results were derived from the last 5 ns trajectory. The density profile was symmetrized around the membrane center to obtain a better result. The density profiles of T4-DM in dry and hydrated states almost overlapped. As for T4-SP and T4-SB membranes, the density profile of hydrated state broadened compared with that of dry state (more obvious for T4-SB membrane), which is consistent with the configurations in Figure S1. The density of water in T4-SB is higher than that of T4-SP, and even higher than that of T4-DM, which indicates that the side chains of T4-SB can attract extra water molecules compared with those of T4-SP and T4-DM. We then deduce that the hydrophilicity of the antifouling membranes follows the order of T4-SB > T4-SP > T4-DM.

#### 3.1.2. Surface Roughness

**Figure 4:**
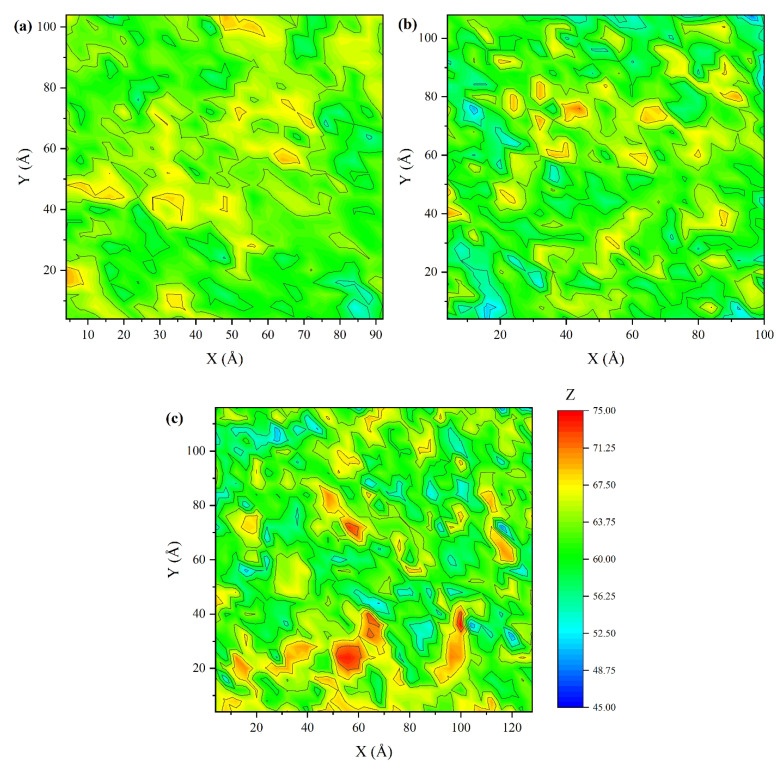
Contour maps of three antifouling membrane surfaces. (**a**) T4-DM, (**b**) T4-SP, (**c**) T4-SB.

**Figure 5:**
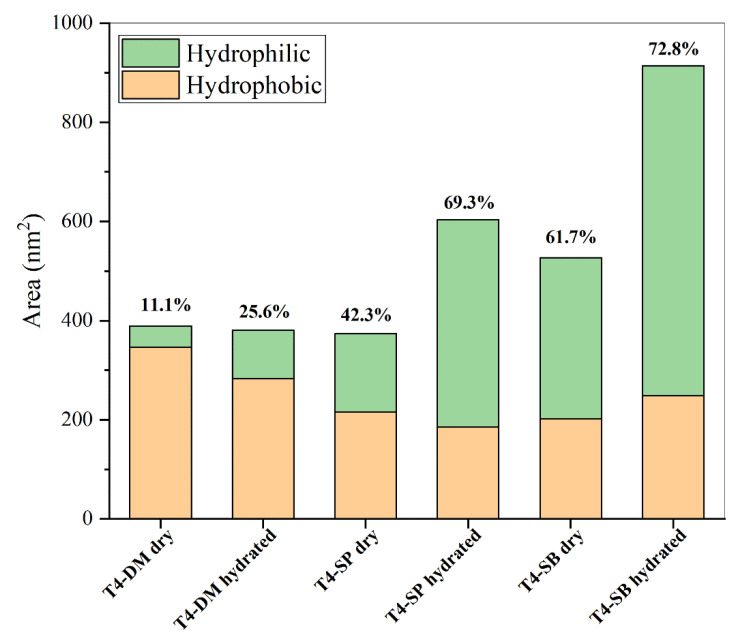
Solvent accessible surface area including hydrophobic and hydrophilic part of three antifouling membranes. Numbers above the bar means the proportion of hydrophilic area.

Since the density profile is a statistical average of the entire membrane layer, it cannot reflect the local specific structural information of membranes. To further analyze the detailed surface structure, contour maps of the upper surface of three antifouling membranes in hydrated states were sketched, as shown in Figure 4. To define the surface of membrane, the simulation box was divided into grids with 0.4 nm × 0.4 nm resolution in xy plane. Atoms with the largest or smallest z-axis were selected as the top atoms to define the membrane surface. It can be seen from Figure 5 that T4-DM membrane’s surface is relatively flat, while T4-SP has more peaks and valleys than T4-DM. As for T4-SB, the contour lines are the densest, indicating that the order of surface roughness is T4-SB > T4-SP > T4-DM.

To quantify the surface roughness of three antifouling membranes, the root mean square roughness R was introduced [37]:

$$R=\sqrt{\frac{\sum_{i=1}^{N}{\left(Z_{i}-\bar{Z}\right)}^{2}}{N}}$$

where *Z_i_* is the z-coordinate of the atoms exposed in the outermost layer in each grid point and $\bar{Z}$ is the average value of the z-coordinates of all the atoms exposed on the outermost surface. Both the up and down surfaces of three antifouling membranes in dry and hydrated states are calculated and listed in Table 1. The data suggested there is little difference between dry and hydrated states for T4-DM. The roughness in hydrated state follows the order of T4-SB > T4-SP > T4-DM, which is consistent with Figure 3 and Figure 5. Obviously, the greater the roughness of the surface, the more hydrophilic sites were exposed, and the more water molecules could be bound.

#### 3.1.3. Hydrophilicity

In addition to the influence of surface roughness on surface hydration, the hydrophilicity and hydrophobicity of the surface determine the surface hydration ability directly. The hydrophilic and hydrophobic surface area of each antifouling membrane were calculated from the last 5 ns trajectory, as shown in Figure 5. During calculation, the atomic charge between −0.2 and 0.2 was considered as the hydrophobic surface area, and the other is the hydrophilic surface area. The hydrophilic surface area and its proportion of all three antifouling membranes increased in hydrated state. The total surface area does not change much between dry and hydrated states, which is consistent with the configuration in Figure S1. The total surface area, especially the hydrophilic surface area, of T4-SP and T4-SB both increased significantly when immersed in water, which suggests that they have a strong hydration ability.

### 3.2. Properties of Surface Hydration Layer

#### 3.2.1. Structural Properties 

**Figure 6:**
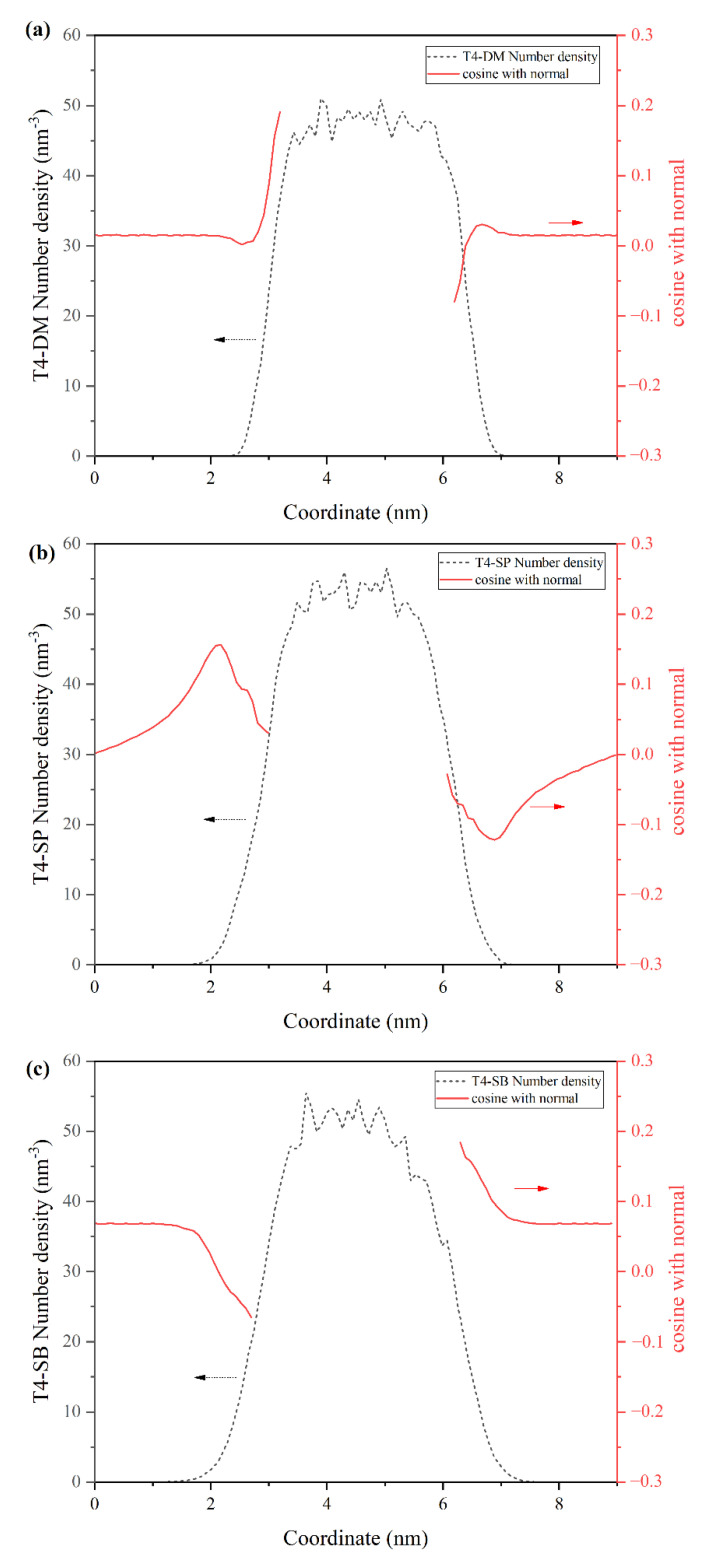
Water dipole orientation profiles of three antifouling membranes. (**a**) T4-DM, (**b**) T4-SP, (**c**) T4-SB.

After the above structural analysis of the antifouling membranes, it was found that the surface hydration ability of the three antifouling membranes was T4-SB > T4-SP > T4-DM. We also noticed that with the increase in surface hydration ability, more water molecules can penetrate into the matrix of membrane from the density profiles in Figure 3. To examine the structure of water molecules near the interface of antifouling membranes, we calculated the cosine of the angle between dipole of water and z-axis at different distances from the surface, as shown in Figure 6. Obviously, for a random distribution, the cos*θ* should be close to 0 [38]. In the T4-DM membrane system, only water molecules close to membrane have a certain orientation, while water molecules farther away are randomly distributed. In the T4-SP system, the dipole orientation of surface water molecules slightly decreased to 0 after 2 nm, while in the T4-SB system, there is still a long-distance arrangement of water molecules even beyond 2 nm away from the surface. This observation is consistent with Leng’s experiment [23,24], where ordered water molecules were found at zwitterionic pSBMA surfaces.

#### 3.2.2. Dynamic Properties

**Figure 7:**
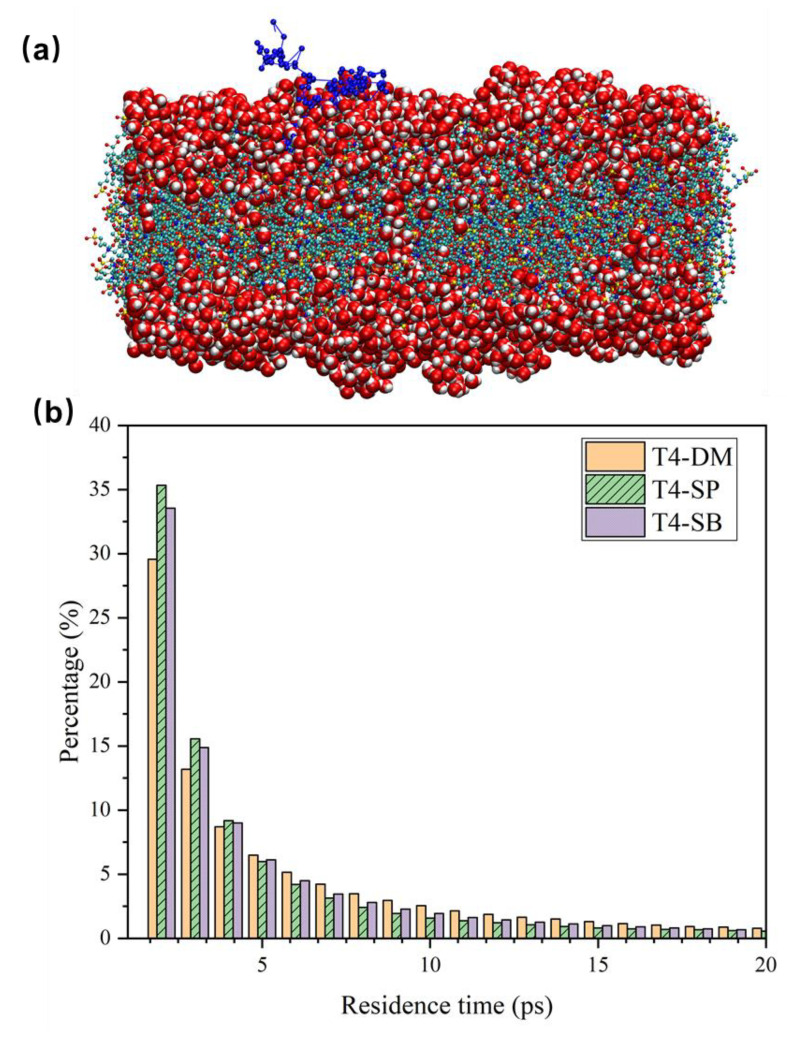
Trajectory and residence time of surface hydration layer water molecules. (**a**) Trajectory of one hydration layer water molecule above T4-SB surface (connected blue dots). The antifouling membrane was colored in CPK mode. The surface hydration layer water molecules were modeled in VDW mode. (**b**) Residence time distribution of water molecules in the hydration layer of three antifouling membranes.

The antifouling membranes can also affect the hydration layer’s dynamic properties beside the structure of surface water molecules. We calculated the distribution of the average residence time of water molecules within 0.5 nm of antifouling membrane surfaces, as shown in Figure 7b. The average residence time means how long water molecules can stay near the surface of the antifouling membrane on average [39]. It reflects the stability of the hydration water layer of the antifouling membrane or, in other words, the hydration ability of antifouling membranes [40]. Figure 7a shows the trajectory of one hydration layer water molecule above T4-SB membrane. The calculated average residence time is shown in Table 2. It can be seen that the average residence time increased from T4-DM and T4-SP to T4-SB, indicating that the binding effect of antifouling membranes on their surface hydration layers increased.

**Figure 8:**
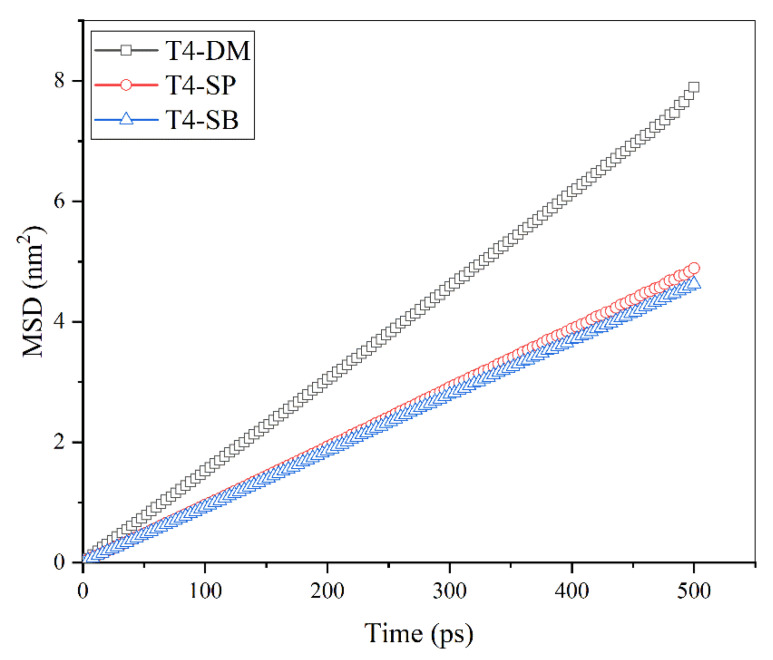
Mean square displacement of water molecules in the hydration layer of nonfouling membrane.

The diffusion behavior of surface hydration layer water molecules above three antifouling membranes was investigated. The mean square displacement (MSD) of surface hydration layer water molecules is shown in Figure 8. Their diffusion coefficients were then calculated according to Einstein’s equation and collected in Table 2. It can be seen that the diffusion coefficients of surface hydration layer water molecules above three antifouling membranes gradually decreased from T4-DM and T4-SP to T4-SB, indicating that the mobility of water molecules decreased or the binding effect from the antifouling membranes increased, which is consistent with the previous analysis.

### 3.3. Hydration Mechanisms—From the View of Monomers

#### 3.3.1. Solvation Free Energy

We have analyzed and compared the structural properties of the antifouling membranes and the structural and dynamic properties of their hydration water layers from the overall antifouling membranes’ view. The order of surface hydration ability or antifouling ability, T4-SB > T4-SP > T4-DM, was obtained. Next, we analyze the mechanisms for the difference in hydration ability from the monomer’s view, which serves as a model for the antifouling polymer membrane [38].

Solvation free energies were calculated for three monomers at M05-2X/6-31 g* level, as collected in Table 3. The negative of solvation free energy indicates all three monomers have a high affinity with water. The order of solvation free energy follows the order of T4-SB > T4-SP >> T4-DM, which is consistent with previous analysis.

#### 3.3.2. Electrostatic Potential

**Figure 9:**
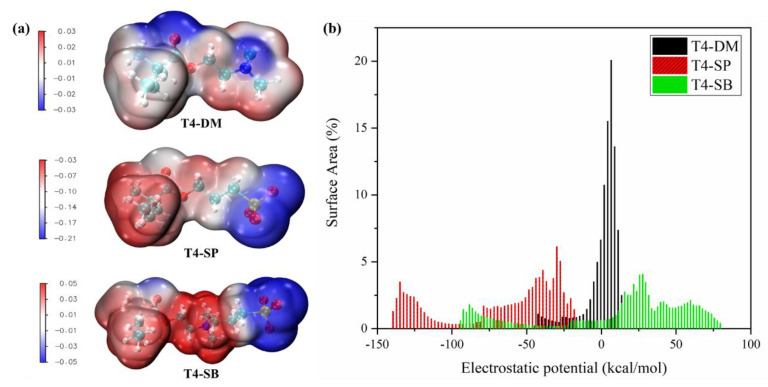
Electrostatic potential of monomers of three antifouling polymers’ monomers. (**a**) Electrostatic potential mapped on vdW surface; (**b**) distribution of surface area percentage of different electrostatic potentials.

Electrostatic potentials of the three monomers were calculated and mapped on their van der Waals surfaces [41], as shown in Figure 9. The molecular polarity, polar, and nonpolar surface area were also calculated, as shown in Table 3 [42]. The surface area with |ESP| <= 10 kcal/mol was considered as nonpolar surface area while the others were considered as polar surface area. It can be seen that the negative charge center of T4-DM monomer is located at the N atom. Since T4-SP monomer has a negative charge, the overall electrostatic surface is negative, and mainly concentrated on the sulfonate group. In the zwitterion T4-SB monomer, the negative charge center is located in the sulfonate group and the positive charge center is located at the N atom. Though the MPI of T4-SP was the largest, the T4-SB has the largest polar surface area, which can combine with more water. Combining with the distribution of areas occupied by different electrostatic potential regions in Figure 9b, it can be seen that the distribution of electrostatic potential on the surface of T4-SB monomer is the broadest, which is conducive to the electrostatic interaction with other polar molecules such as water [43].

#### 3.3.3. Radial Distribution Function

**Figure 10:**
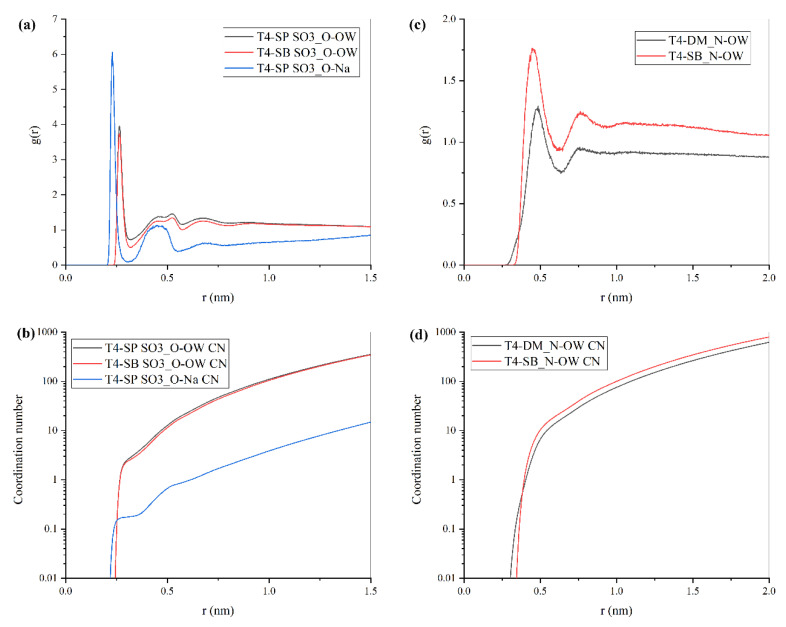
Radial distribution functions (RDFs), g(r) of hydration groups on antifouling membrane surface, and the cumulative number. (**a**) RDF of SO_3_^−^_O-OW and SO_3_^−^_O-Na^+^, oxygens in SO_3_^−^ groups as referenced atoms. (**b**) RDF of N-OW, nitrogen atoms as referenced atoms. (**c**) Cumulative number of SO_3_^−^_O-OW and SO_3_^−^_O-Na^+^. (**d**) Cumulative number of N-OW.

**Figure 11:**
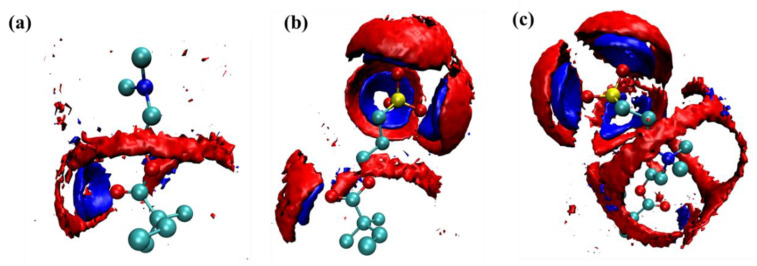
Spatial distribution function of water molecules around three different antifouling monomers. (**a**) DM, (**b**) SP, (**c**) SB.

To further understand the hydration ability of antifouling polymers’ monomers, another molecular dynamics simulation was conducted. Three monomers were solvated in 4 × 4 × 4 nm^3^ water box, respectively; then, 50 ns NPT simulations were performed. After that, the radial distribution functions (RDFs) of the water molecules or Na^+^ around the polar groups of three monomers and their coordination number were calculated, respectively, as shown in Figure 10. The RDFs can reflect the intermolecular structure and interactions between center atoms and surrounding water molecules. Two peaks were found in the RDF curve, indicating that two hydration layers were formed, which corresponded to the first hydration layer that consisted of bound water and the second hydration layer made up of trapped water; this agrees with Paul’s experiment [25]. According to Figure 10a,b, SO_3_^−^ groups in T4-SP and T4-SB have similar hydration ability and are stronger than the N group in T4-DM and T4-SB. Meanwhile, the peaks of g(r)_N-OW_ in T4-DM were lower than those in T4-SB and also the coordination number of the first hydration shell from Figure 11c,d, indicating a better packed hydration shell around N in T4-SB. The number of water molecules tightly bonded to three monomers were also calculated and collected in Table 3. Consequently, the T4-SB antifouling membrane presents a more hydrophilic behavior than T4-SP and T4-DM.

#### 3.3.4. Spatial Distribution Function

Though the RDFs can reflect the hydration effect of hydrophilic groups in three monomers on water molecules, the calculation of RDFs is based on the spherical averaging of the water molecules around the hydrophilic group, which neglects the spatial distribution of the water molecules. Therefore, the spatial distribution function (SDF) of water molecules around hydrophilic groups was calculated, shown in Figure 11. From this, we can see that there is only a ribbonlike distribution around the carbonyl oxygen in DM monomer, while the distribution of water molecules around the N atom cannot be shown under current isosurface. In the SP monomer, there are three spherical crown water molecule distribution areas in the direction of three S–O bonds, which is obviously caused by the hydrogen bond formed between the O atom in SO_3_^−^ group and the water molecules. Similar structures were also found in SB monomer. Besides this, there is a ribbonlike distribution of water molecules around the N atom.

#### 3.3.5. Noncovalent Interactions

**Figure 12:**
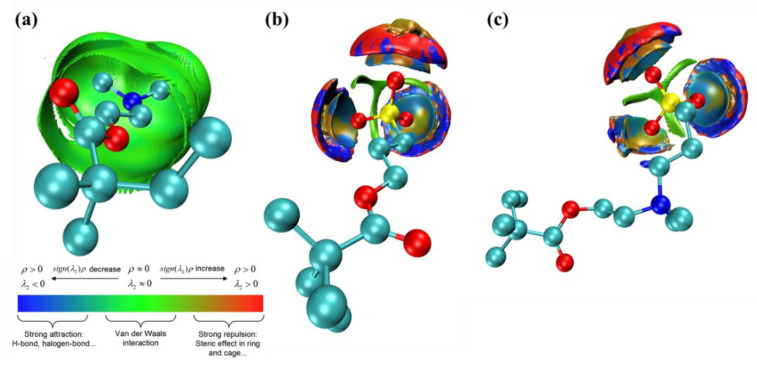
Noncovalent interaction around three different nonfouling repeat units. (**a**) DM, (**b**) SP, (**c**) SB.

To fundamentally understand the different hydration ability of three antifouling monomers, aNCI (averaged noncovalent interaction) analysis [44,45] was conducted, shown in Figure 12. The green area in the figure indicates that van der Waals interaction is dominant. Blue area indicates that there is a strong hydrogen bond interaction. The red area indicates that there is a strong steric hindrance effect. In DM monomer, as the negative charge center N atom was shielded by surrounding methyl groups, it can only interact with water molecules through weak vdW interactions. In T4-SP and T4-SB monomers, water molecules can directly form hydrogen bonds with the exposed O atoms, which plays a key role in their strong hydration ability. Besides that, the extra positive charge center N atom can also interact with water molecules through weak vdW interactions such as N in the T4-DM monomer. Therefore, the hydration abilities of three antifouling polymers are in the order of T4-SB > T4-SP > T4-DM.

## 4. Conclusions

In this work, we investigated the surface hydration of three antifouling membranes—T4-DM, T4-SP, and T4-SB—by a series of molecular dynamics simulations. Dipole orientation distribution, diffusion coefficient, and average residence time revealed an orderly, packed, and tightly bound surface hydration layer above T4-SP and T4-SB antifouling membranes. The surface structure, density profile, surface roughness, and area percentage of hydrophilic surface provide further details regarding the strong surface hydration of T4-SP and T4-SB from the membranes’ aspect. The side chains of T4-SP and T4-SB were more stretched in hydrated state due to their high hydration ability, which can cause steric repulsion and prevent adsorption. Their surfaces are relatively rough, which can bind much more water or even let water penetrate into the internal voids of the membrane.

To further understand the surface hydration ability of three antifouling membranes, solvation free energy, electrostatic potential, RDFs, SDFs, and noncovalent interactions of three monomers were analyzed. T4-SB monomer has the broadest distribution of electrostatic potential on the surface, resulting from the separated negatively and positively charge center and largest water coordination number for its zwitterionic architecture. Its exposed negative charge center SO_3_^−^ group can form hydrogen bonds with surrounding water molecules and the shielded positive charge center N can also bind water molecules through weak vdW interaction.

The simulation data suggest the hydration ability of monomers ranks in terms of T4-SB > T4-SP > T4-DM. Since the surface hydration layer serves as a physical and energy barrier during the prevention of protein adsorption, we believe their antifouling ability ranks in terms of T4-SB > T4-SP > T4-DM, which is consistent with experiments.

## Figures and Tables

**Table 1:** Root-mean-square roughness of three antifouling membranes.

	Root-Mean-Square Roughness R *			
	Hydrated		Dry	
	Up	Down	Up	Down
T4-DM	2.80 ± 0.074	2.84 ± 0.062	2.79 ± 0.056	2.80 ± 0.068
T4-SP	3.73 ± 0.050	3.83 ± 0.056	2.68 ± 0.037	2.41 ± 0.037
T4-SB	4.28 ± 0.068	4.20 ± 0.059	2.94 ± 0.042	2.98 ± 0.032

* Data derived from the last 1 ns trajectory.

**Table 2:** Dynamic properties of hydration layer water molecules above three antifouling membranes including average residence time and diffusion coefficient.

Antifouling Membranes	Average Residence Time (ps)	Diffusion Coefficient D × 10^−5^ cm^2^/s
T4-DM	17.85	2.57 (+/− 0.080)
T4-SP	24.98	1.62 (+/− 0.014)
T4-SB	27.16	1.54 (+/− 0.043)
Bulk water	-	4.13(+/− 0.15)

**Table 3:** Properties of monomer of three antifouling membranes.

Monomer of Antifouling Membranes	Solvation Free Energy (kcal/mol)	Nonpolar Surface Area (Å^2^)	Polar Surface Area (Å^2^)	Molecular Polarity Index (kcal/mol)	Number of Bonded Water Molecules
T4-DM	−6.16	203.59	63.16	8.58	10.02
T4-SP	−71.68	0.00	283.56	67.07	15.43
T4-SB	−73.46	24.60	339.35	43.54	18.43

## Data Availability

Not applicable.

## Supplementary Materials

The following supporting information can be downloaded at: https://www.mdpi.com/article/10.3390/molecules27103074/s1, Figure S1: Final simulation configuration of three antifouling membranes under dry and hydrated states; Table S1: 21-step MD compression and relaxation schemes.

## Sample Availability

Samples of the compounds are not available from the authors.
