# Supplementary material for: Molecular Dynamics Study on Properties of Hydration Layers above Polymer Antifouling Membranes
Source: Molecules. 2022 May 11;27(10):3074. doi: 10.3390/molecules27103074 (PMC9143230; doi:10.3390/molecules27103074)
Supplement: Supplementary file 1 [file molecules-27-03074-s001.zip › molecules-1695802-supplementary.pdf]

Supporting Information for

Molecular dynamics study on properties of hydration layers above polymer antifouling membranes

Heng Zhang[†], Jiyong Zheng[‡], Cunguo Lin[‡], Shiling Yuan[*†]

† *Key Lab of Colloid and Interface Chemistry, Shandong University, Jinan 250199, P. R. China*

‡ *State Key Laboratory for Marine Corrosion and Protection, China Shipbuilding Industry 725 Research Institute, Qingdao 266071, P. R. China*

Table S1 21-step MD compression and relaxation schemes

Step	Simulation details	Simulation time (ps)
1,2	NVT 600 K，NVT 300 K	50,50
3	NPT 0.02P_{max} bar, 300 K	50
4,5	NVT 600 K，NVT 300 K	50,100
6	NPT 0.6 P_{max} bar, 300 K	50
7,8	NVT 600 K，NVT 300 K	50,100
9	NPT P_{max} bar, 300 K	50
10,11	NVT 600 K，NVT 300 K	50,100
12	NPT 0.5 P_{max} bar, 300 K	5
13,14	NVT 600 K，NVT 300 K	5,10
15	NPT 0.1 P_{max} bar, 300 K	5
16,17	NVT 600 K，NVT 300 K	5,10
18	NPT 0.01 P_{max} bar, 300 K	5
19,20	NVT 600 K，NVT 300 K	5,10
21	NPT 1 bar, 300 K	50000

*shilingyuan@sdu.edu.cn

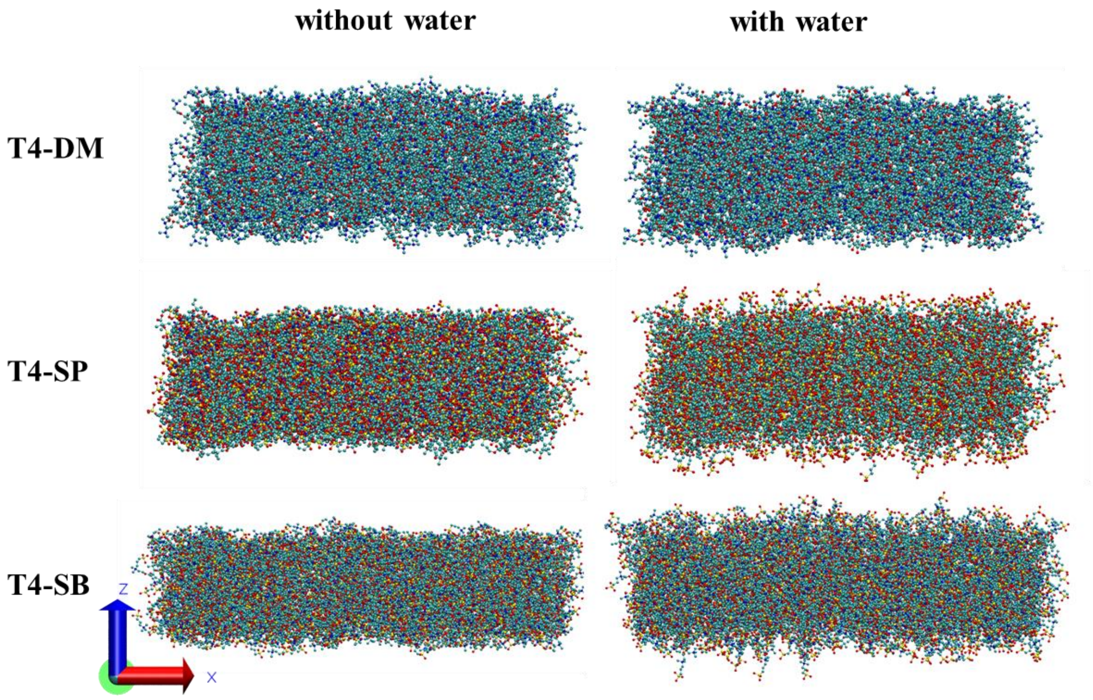

Figure S1 Final simulation configuration of three antifouling membranes under dry and hydrated states. Water molecules were not shown for clarity.
